# A Review of Electroactive Nanomaterials in the Detection of Nitrogen-Containing Organic Compounds and Future Applications

**DOI:** 10.3390/bios13110989

**Published:** 2023-11-18

**Authors:** Mohanraj Jagannathan, Durgalakshmi Dhinasekaran, Ajay Rakkesh Rajendran, Sungbo Cho

**Affiliations:** 1 Department of Electronic Engineering, Gachon University, Seongnam-si 13210, Republic of Korea; mohanrajpsix@gmail.com; 2 Department of Medical Physics, College of Engineering Campus, Anna University, Chennai 600 025, Tamil Nadu, India; durgalakshmi@annauniv.edu; 3 Functional Nano-Materials (FuN) Laboratory, Department of Physics and Nanotechnology, Faculty of Engineering and Technology, SRM Institute of Science and Technology, Kattankulathur 603 203, Tamil Nadu, India; ajayr1@srmist.edu.in; 4 Gachon Advanced Institute for Health Science & Technology, Gachon University, Incheon 21999, Republic of Korea

**Keywords:** nanomaterials, electrochemical, impedimetric, nitrogen–containing organic compounds, sensors

## Abstract

Electrochemical and impedimetric detection of nitrogen-containing organic compounds (NOCs) in blood, urine, sweat, and saliva is widely used in clinical diagnosis. NOC detection is used to identify illnesses such as chronic kidney disease (CKD), end-stage renal disease (ESRD), cardiovascular complications, diabetes, cancer, and others. In recent years, nanomaterials have shown significant potential in the detection of NOCs using electrochemical and impedimetric sensors. This potential is due to the higher surface area, porous nature, and functional groups of nanomaterials, which can aid in improving the sensing performance with inexpensive, direct, and quick-time processing methods. In this review, we discuss nanomaterials, such as metal oxides, graphene nanostructures, and their nanocomposites, for the detection of NOCs. Notably, researchers have considered nanocomposite-based devices, such as a field effect transistor (FET) and printed electrodes, for the detection of NOCs. In this review, we emphasize the significant importance of electrochemical and impedimetric methods in the detection of NOCs, which typically show higher sensitivity and selectivity. So, these methods will open a new way to make embeddable electrodes for point-of-detection (POD) devices. These devices could be used in the next generation of non-invasive analysis for biomedical and clinical applications. This review also summarizes recent state-of-the-art technology for the development of sensors for on-site monitoring and disease diagnosis at an earlier stage.

## 1. Overview of Urea Analysis in Ancient Eras

**Figure 1:**
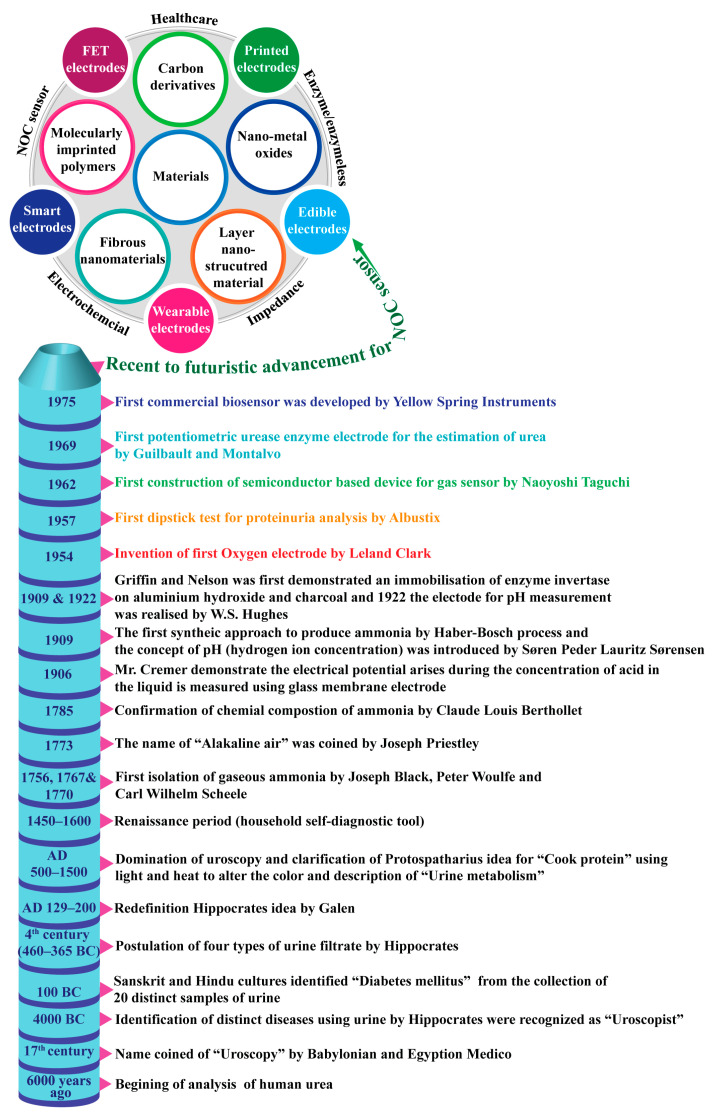
Historical evidence related to the conceptualization, differentiation, and development of nanomaterials for the detection of nitrogen-containing organic compounds.

Over 6000 years ago, diagnostics were first used as an element of laboratory practice. Urine is an inexpensive source for finding nitrogen-containing organic compounds (NOCs) and demonstrates their potential correlation with various illnesses. This excretory biofluid was a primary diagnostic tool in ancient eras and was considered a “divine fluid” by physicians. Babylonian and Egyptian medicos coined the word “uroscopy”, which was derived from the Greek “ouron” (i.e., denoting “urine”) and “skopeo” (i.e., denoting “examine or inspect”). In 100 B.C., Sanskrit and Hindu cultures classified urine into twenty distinct types based on its color, and it was further named by its sweet taste in a special case called diabetes mellitus [1]. In the 4th century B.C. (460–365), Hippocrates found the presence of filtrates, such as blood, phlegm, and yellow and black bile, in urine [2]. The predominant theory of Hippocrates’ ideas were further redefined precisely by Galen (AD 129–200), followed by a redefinition in the Middle Ages (AD 500–1500), whereby uroscopy played a vital role in all the stages of disease identification [3,4,5]. Then, in the Renaissance period (AD 1450–1600), uroscopy was developed as a household self-diagnostic tool for monitoring health conditions. Nevertheless, ancient techniques of diagnosis with urine are not practiced nowadays, yet urine remains an effective tool for the early detection of diseases in the modern world [4]. Figure 1 shows the historical evidence related to the conceptualization, differentiation, and development of nanomaterials for the detection of nitrogen-containing organic compounds.

## 2. Introduction

**Figure 2:**
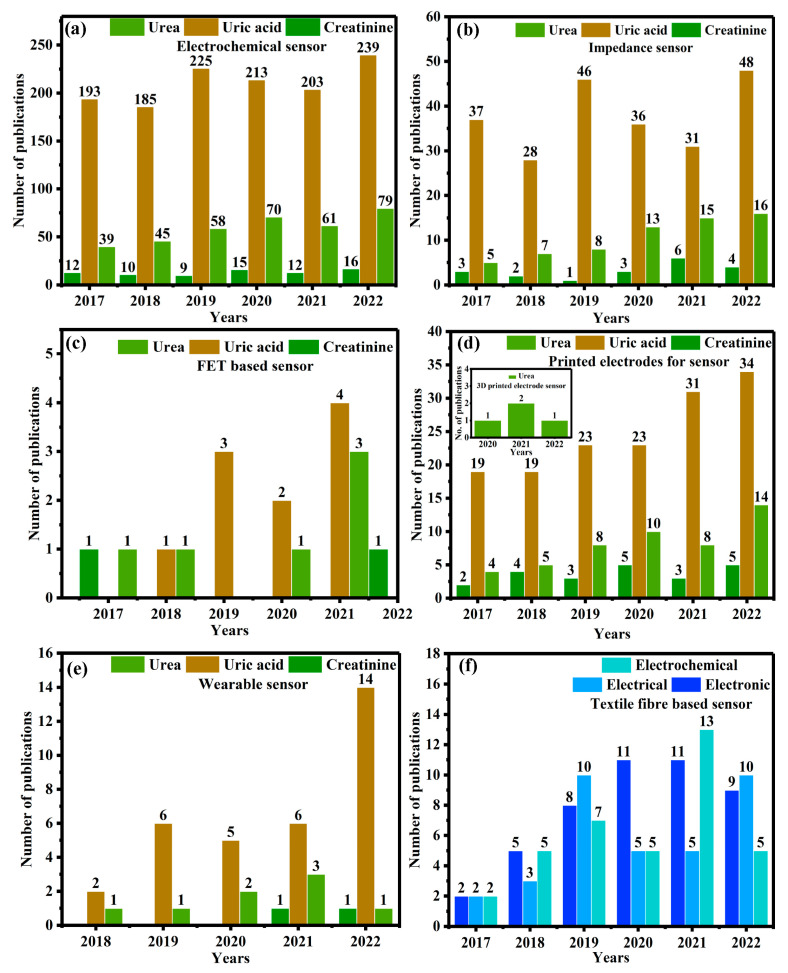
The Scopus index bar chart is related to the number of publications on the topics of (**a**) electrochemical sensors, (**b**) impedimetric sensors, (**c**) FET-based sensors, (**d**) printed electrode sensors, (**e**) wearable sensors, and (**f**) textile fiber-based sensors.

In recent decades, the sustainable economic progress of public health has been dependent on the advancement of science and technology. Therefore, the scientific community has predicted the necessities for the development of effective ideal materials, methodologies, and technology transfers. Despite this, researchers are facing many challenges in treating various illnesses (i.e., cancer, diabetes, neurological dysfunction and/or disorder, etc.), managing food safety and quality, and environmental remediation. Typically, nitrogen-containing organic compounds (NOC) (i.e., urea, uric acid, and creatinine) are naturally found in biofluids such as blood, urine, saliva, and sweat [6]. These NOCs are effectively filtered through the kidneys and liver while also being discreetly filtered through the biodegradation of food as well as environmental systems [7]. In addition, the determination of NOCs is an important biomarker for clinical diagnosis and socio-environmental monitoring [8]. Particularly in clinical diagnosis, urine and blood are considered precious tools for identifying liver and kidney function, urinary tract obstruction, heart failure, catabolism of proteins, and even shock and stress [9]. Table 1 and Table 2 show the normal NOC levels of urea, uric acid, and creatinine in biofluid. Increasing or decreasing NOC levels can cause various illnesses. Therefore, extensive research on NOCs poses great challenges in terms of precise determination via analytical methods, even in the presence of complex metabolites at a lower concentration. Of these, an enormous number of conventional methods are being used to identify NOCs, including solid-phase extraction (SPE), ultraviolet-visible (UV-visible) spectroscopy, infrared (IR) spectroscopy, flow injection, chromatography techniques coupled with mass spectrometry, fluorimetry, and surface plasmon resonance (SPR) techniques [10,11,12,13]. Furthermore, these methods necessitate sophisticated instrumentation, trained individuals, a time-consuming process, pre-sample preparation, etc. Despite this, electrochemical/electroanalytic techniques have shown promise for direct and indirect quantitative and qualitative determination of NOCs, with good sensitivity, selectivity, a rapid response, and feasible economic viability [14,15]. This is due to the fact that biochemical events are converted into electrical signals through the electrochemical biosensor, which is a typical sensing device. Generally, electrochemical techniques have been recognized based on their operational conditions, such as amperometry (i–t), differential pulse voltammetry (DPV), linear pulse voltammetry (LSV), square wave voltammetry (SWV), the current-potential (I–V), and electrochemical impedimetric spectroscopy (EIS). Of these, the EIS method is a quick, non-destructive technique that has recently been used to determine NOCs. Briefly, the EIS involves applying an alternating current at various frequencies to the sample, followed by measuring the impedance response from the sample as a function of frequency. Rather than conventional techniques, the affordable technique of voltametric measurement has been used to determine the redox potential, whereas the potentiometric method is employed to measure precision, selectivity, and sensitivity. Figure 2 depicts the Scopus index bar chart on the number of research publications based on NOC biomarker detection from the period from 2017 to 2022 and towards the future innovation and sustainable development of wearable devices.

**Figure 3:**
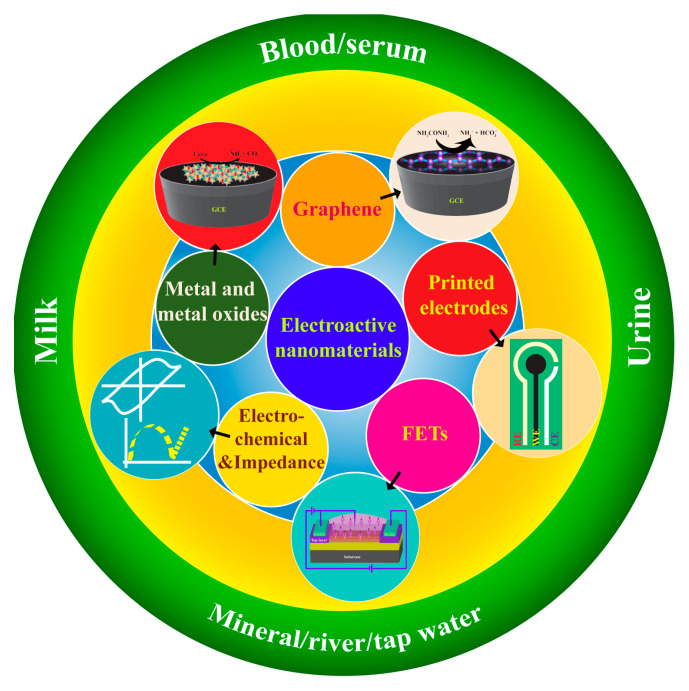
A schematic of the diverse range of NOC sensors that use nanomaterials and their fabricated devices.

Electrochemical methods are used to determine NOC levels in serum, blood, sweat, and urine, and are of great interest to researchers due to their rapid accessibility. In principle, it is necessary to detect NOC levels via either enzymatic or non-enzymatic electrochemical methods. Furthermore, the enzymatic electroanalytical technique is an indirect method that uses enzymes (i.e., urease), which is referred to as an “enzymatic electrochemical sensor”, and vice versa for the non-enzymatic method. These enzymatic or non-enzymatic electroanalytical sensors have certain inevitable drawbacks, as follows: immobilization of enzymes; expensive enzyme; incompetent reproducibility; and limited concentration, temperature, pH, and humidity conditions [10,23,24,25,26,27,28]. On the other hand, development of biomedical devices for rapid, sensitive, and specific detection of biomarker species has been facilitated by the emerging field of nanomaterials and nanofabrication technology. Indeed, the construction and development of integrated nanosensors for the simultaneous detection of a variety of target analytes will remain a major task on the frontier of nanotechnology [29]. Therefore, the state-of-the-art technology in the manufacture of nano-based devices pays great attention to real-time applications, especially in on-site health monitoring products. The characteristics of mesostructured or composite/hybrid nanostructures are distinctly different from those of conventional bulk materials. Usually, the surfaces of a nanoengineered smart material would have been changed to include functionalities that create binding sites for biomolecules [30]. Then, these nanomaterials were found to have many uses, especially in the development of direct or indirect electrochemical biosensing devices, which are used to measure NOCs at ultra-low concentrations using very small volumes of different clinical samples (i.e., biofluids). Furthermore, the qualitative and quantitative estimation of NOCs has gained immense importance as it offers early diagnosis of several diseases like renal insufficiency, hyperpyrexia, hyperthyroidism, leukemia, diarrheal diseases, diabetes mellitus, and so on [22,31,32]. So, in this review, we have summarized the recent advances in NOC biomarker detection methods through direct or indirect electrochemical and impedimetric-based sensing methods. A wide range of metal oxide nanoparticles and two-dimensional (2D) materials and their nanocomposites have seen considerable progress in their preparation, processing, characterization, and potential applications (Figure 3). In addition, nanofabrication of devices like FETs and printed electronics has resulted in improvements in their performance. Consequently, it is possible to introduce new opportunities in the development of nanoscale devices and make them economically scalable for next-generation sensors, not only biosensors but also sensors in food and environmental monitoring.

## 3. Nanomaterial-Based Electrochemical Biosensors

Electrochemical biosensors, with their advantages of affordability, and rapid processing and construction, have been extensively used in clinical diagnosis, environmental monitoring, pharmaceutical analysis, and onsite detection of home care products [33]. Integration of electrochemical measurements like amperometry, cyclic voltammetry, electrochemiluminescence, impedimetric, and photoelectrochemistry with recognition units, shows significant advantages during the measurement [34]. These electroanalytical techniques have vast applications, including the determination of excretory metabolites [35], metal ions [36], protein biomarkers [37], DNA [38], neurological disorders [39], etc. However, there are still challenges in quantifying low-quantity biomarkers accurately in a complex system, as it requires precise measurement techniques and material fabrication technology. To overcome these limitations, several functional nanomaterials have been developed in recent decades. Additionally, these nanomaterials can be used as electrocatalysts and can therefore amplify the signal by precise changes upon their measurement [40,41]. However, there is an immediate need for the advancement of nanocatalysts with high performance for the construction and development of next-generation non-invasive electrochemical biosensors.

### 3.1. Nickel and Its Nanocomposites for NOC Sensing

**Figure 4:**
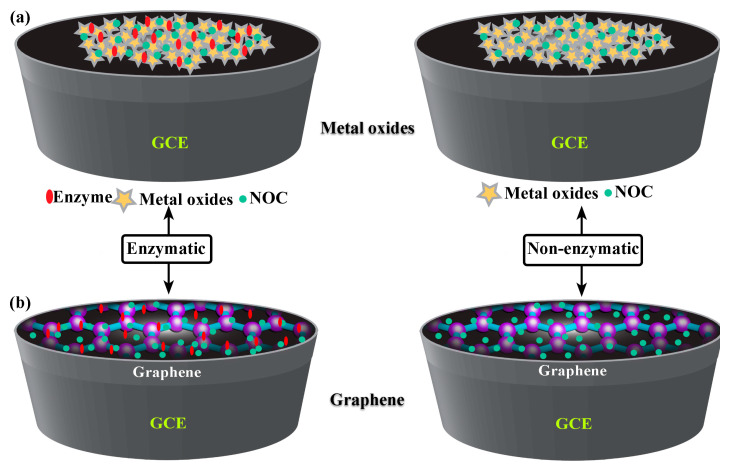
Schematic illustration of the development of (**a**) metal oxides and (**b**) two-dimensional nanomaterials on GCE for the detection of NOCs with enzymatic and enzyme-free methods.

**Figure 5:**
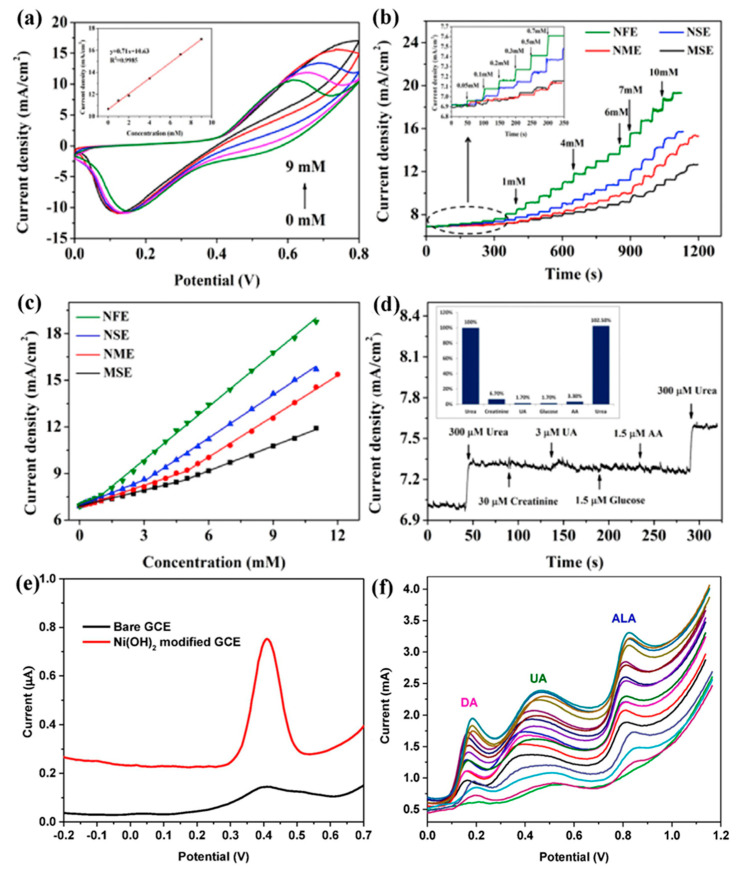
CV profiles of the prepared Ni-P paper electrode (**a**) at different concentrations of urea with a scan rate of 5 mV/s; inset is the plot of oxidation peak currents vs. concentrations of urea, (**b**) amperometric responses of four Ni-P paper electrodes against successive injections of urea, (**c**) calibration curve vs. urea concentrations corresponding to the responses, (**d**) current response to the addition of urea and different interfering species. Reprinted with permission from reference [50] and the corresponding copyright is 2019 Elsevier, (**e**) DPV curve showing the current obtained with bare GCE and Ni(OH)_2_ modified GCE as the working electrode in a 1 mM solution of UA, and (**f**) DPV curves of Ni(OH)_2_ nanosheets for simultaneous detection of UA at different concentrations. Reprinted with permission from reference [56] and the corresponding copyright is 2022 Elsevier.

Transition metal oxides have been extensively used to develop enzymatic/non-enzymatic electrochemical sensors. Metal oxides are highly stable in the ambient atmosphere as well as in an alkaline medium, and the synthesis of metal-based nitride, carbides, and phosphides requires highly sophisticated instruments [42,43]. Therefore, transition metal oxides are more favorable for enzymatic/non-enzymatic electrochemical and impedimetric detection of NOC. At the nanoscale, the properties of metal oxides may be quite different from those of bulk metal oxides. This is because nanoscale metal oxides have a larger surface area, a small size, and an ordered crystalline structure. In addition, the nanoscale metal oxides can also have hierarchical nanostructures, which could improve the ability of these metal oxides to act as electrocatalysts [44]. Furthermore, the transition metal-based oxides could favor several oxidative states as they enable the detection of target analytes via surface functionalization [45]. Among the numerous metal oxides, nickel nanoparticles and their composites have received great attention for NOC detection. Also, introducing a functional group onto their surfaces further enhances their the molecular-level recognition and/or interaction. Hence, these oxides and their composites possess features such as easy fabrication; controllable shape and size; biocompatibility; catalytic, optical, and electrical properties; strong absorption and stability; and outstanding electron-transfer kinetics [46,47]. With these characteristic features, any electroactive nanomaterial can be used in energy conversion and storage, catalysis, drug delivery systems, and especially chemical and biological sensors [48]. In Figure 4a, nickel-based metal oxides are shown to have good stability, low cost, less toxicity, and good electrical conductivity, as they lead to immobilization, rapid transduction, and signal amplification. Also, the high isoelectric point (i.e., IEP: 9 to 11) of nickel-based metal oxide possesses the ability to enhance the physical adsorption of biomolecules on these metal oxides through electrostatic interactions [49]. Recently, L. Zheng et al. successfully used a method of electroless deposition for the synthesis of Ni-P nanostructures on a paper substrate, which was used for the detection of urea. Here, paper substrates facilitated the highest surface area for electroless deposition of Ni-P nanoflowers, resulting in high electrocatalytic activity for urea detection (Figure 5a–d). A real sample of swimming pool water was used to determine the urea level in this study. Notably, this device has a broad linear range (0 to 1 mM) with a low detection limit (12 μM), higher sensitivity (683.46 μA mM^−1^ cm^−2^), and quick response time (3 s) [50]. In an alkaline medium, R. H. Tammam and M. M. Saleh studied identical NiOx electrodeposited on a glassy carbon electrode (GCE), which showed better urea oxidation. Further, the corresponding EIS (electrochemical impedimetric spectroscopy) equivalent circuit of the material with a Nyquist plot showed semicircle fitting because of its higher electrocatalytic rates with diffusion-controlled irreversible processes, leading to a lower charge transfer rate [51]. Electrocatalysts like nickel microwire-intercalated cobalt zeolitic imidazole framework (Co-ZIF) without enzymes can be used for the rapid detection of urea. The one-pot solvothermal method was used to make Co-ZIF-nickel nanowires, and the GCE surface was modified with the drop casting method. Arul et al. found that the electrocatalytic oxidation of urea was carried out in the presence of different electrolytes and the composite modified GCE. However, the reaction was more catalytic in Tris-HCl (pH 8.0) buffer solutions than in KOH, acetate, and phosphate buffer solutions, and the way the reaction happened showed that it was controlled by diffusion. Therefore, these authors showed the determination of urea in real samples and the determination was cross validated via the enzymatic method [52]. NiO immobilized on carbonized eggshell (NiO/c-ESM) has also been shown to be a promising material for electrochemically detecting urea in an environmentally friendly way. Lu, S. et al. made a NiO/c-ESM modified with GCE which was used for the electrocatalytic oxidation and reduction of urea in a KOH medium with a significant linear range and limit of detection. CV and square wave voltammetry have were in this study, and it is clear that the oxidation and reduction follow a typical diffusion-controlled process. This process is evident because the NiO/c-ESM system exposes more active sites, which makes it easier for reactants and products to determine urea [53]. Goda, M. A. et al. have also shown that electrochemical deposition can be used to make CuOx-NiOx nanocatalysts with polyaniline/GCE surfaces. So, the NiOx/CuOx/PANI/GCE modified electrode showed better and more stable electrocatalytic performance than the NiOx/CuOx/GCE electrode, as well as easy electron transfer kinetics for urea oxidation [54]. A low-temperature growth method is used for the synthesis of NiCo_2_O_4_ nanoneedles towards the development of enzyme-less detection of urea. Amin, S. et al. proposed non-enzymatic detection of urea in the presence of an alkaline medium and observed that both Ni^2+^ and CO^2+^ oxidized into Ni^3+^ and CO^3+^ after the adsorption of OH^−^ions. Thereafter, urea is adsorbed onto the NiOOH via Ni-O and O-C coordinate linkages, subsequently leading to the direct oxidation of urea, which can take place on the NiOOH and cause the reduced form of Ni(OH)_2_. Also, the cobalt ions couldn’t significantly favor the oxidation of urea, which is due to the presence of Co^4+/^Co^3+^ active sites [55]. In another work, Tomy, A. M., demonstrated nickel hydroxide nanosheets, which were prepared by the facile co-precipitation method and dropped onto the GCE (Figure 5e,f). The possible sensing mechanism of this electrode exhibited that the amine group in uric acid can bond with the hydroxyl group in the sensor molecule, resulting in the possibility of electron transfer and oxidation of UA to allantol [56]. A detailed comparison of the literature related to NOC sensors using metal oxides is given in Table 3.

### 3.2. Graphene and Its Nanocomposites for NOC Sensing

**Figure 6:**
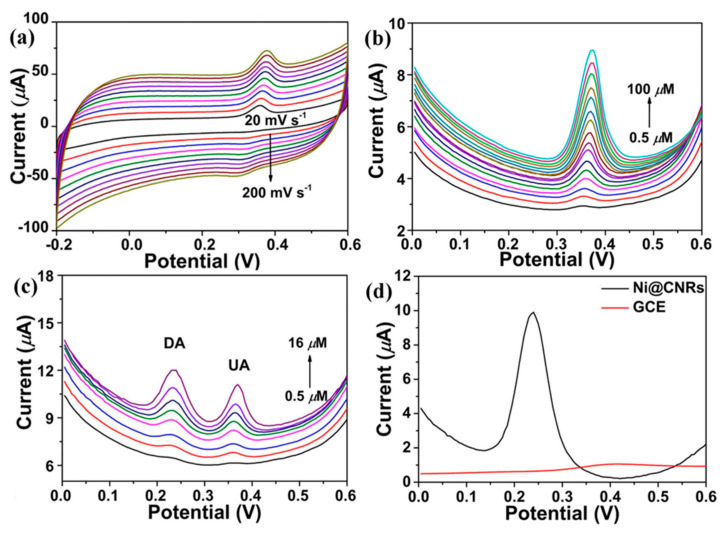
CV curves of Ni@CNRs@GCE with (**a**) 100 μM UA with different scan rates, (**b**) DPV curves with different concentrations, (**c**) simultaneous detection of UA with different concentrations, and (**d**) 100 μM UA. Reprinted with permission from reference [65] and the corresponding copyright is 2021 Elsevier.

**Figure 7:**
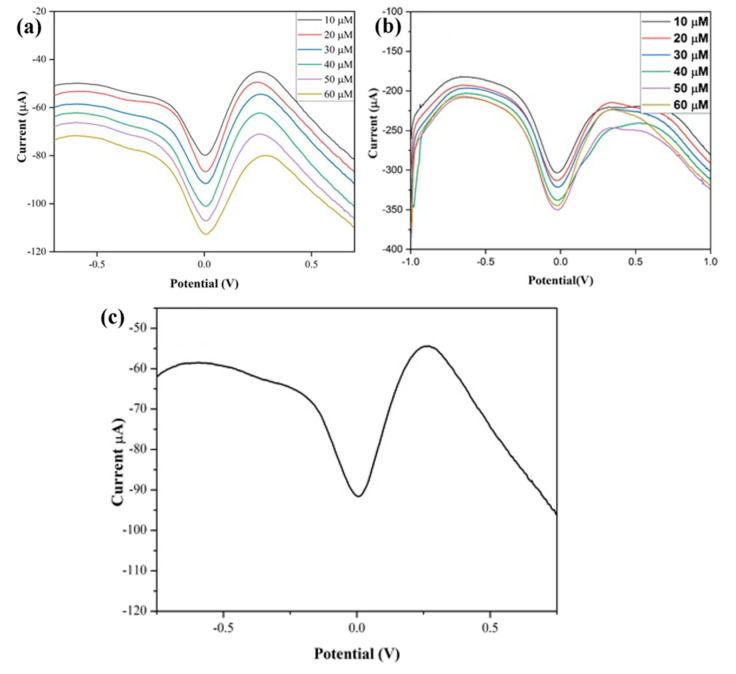
DPV of Ni/RGO/CCF (**a**) at different concentrations of UA, (**b**) on a real sample of human sweat with known urea concentrations, and (**c**) with UA in 20 μL sweat. Reprinted with permission from reference [66] and the corresponding copyright is 2022 Elsevier.

**Figure 8:**
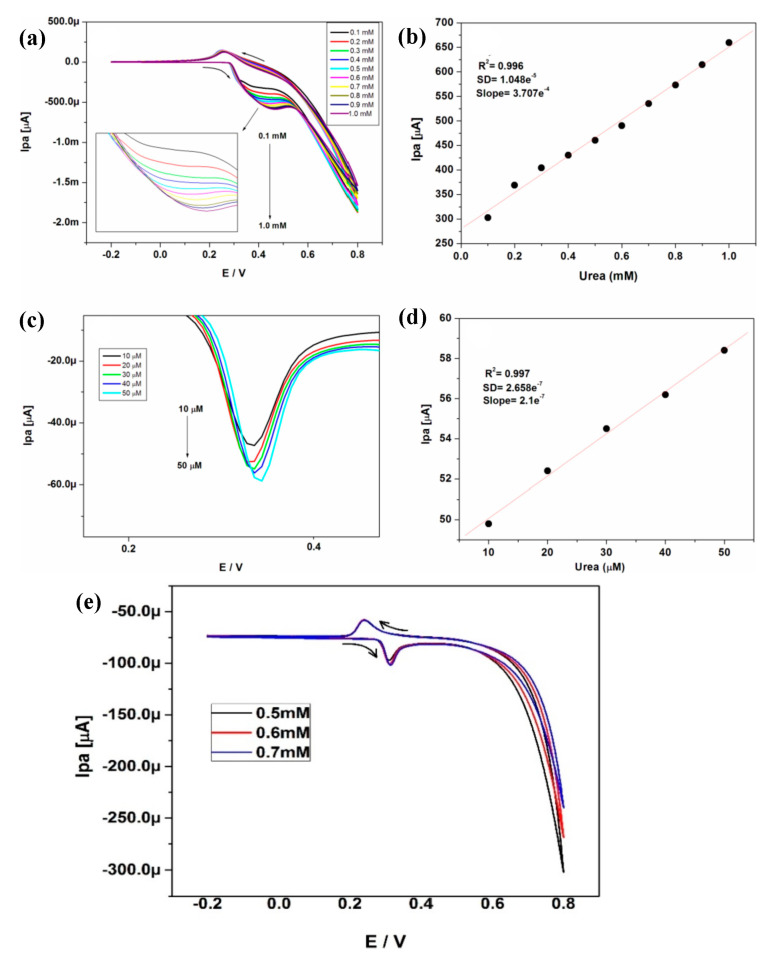
(**a**) CV curve for various urea concentrations (0.1–1.0 mM) detected using NiS/GO/MGCE, (**b**) anodic peak current against concentration, (**c**) DPV curve for urea at different concentrations (10–50 μM), (**d**) anodic peak current against concentration, and (**e**) CV curve of a milk sample spiked with different concentrations of urea on NiS/GO/MGCE. Reprinted with permission from reference [67] and the corresponding copyright is 2020 Elsevier.

**Figure 9:**
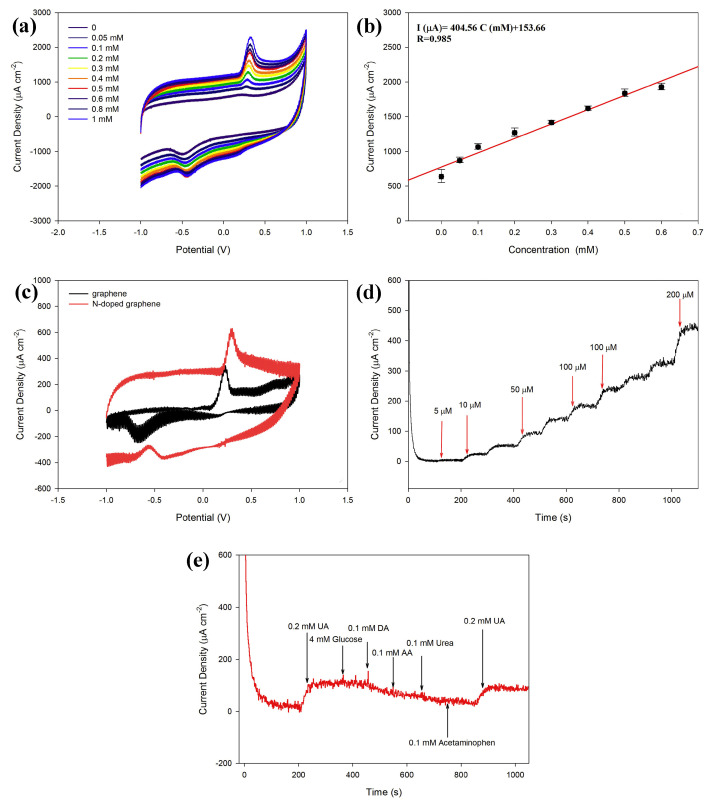
(**a**) CV curve of the N-doped graphene electrode at different concentrations of UA, (**b**) linear fit of current vs. concentration of UA, (**c**) CV curve of the neat graphene and N-doped graphene electrodes after subtracting the background in 0.1 M phosphate buffer (PB) containing 0.2 mM UA, (**d**) amperometric response of the N-doped graphene modified electrode with subsequent infusion of various concentrations of UA, and (**e**) amperometric response of the N-doped graphene electrode toward UA under the effect of different interferents. Reprinted with permission from reference [72] and the corresponding copyright is 2018 Elsevier.

In the last few decades, there has been an increase in the use of multifunctional two-dimensional nanostructure materials, which have the potential to attract exceptional attention due to their physical, chemical, mechanical, and electrical properties. The first successful micromechanical exfoliating method for single-layer graphene was developed by Geim and Novoselov in 2004 [61]. However, certain features, like the number of stacked layers existing in the exfoliation, determine the bandgap and conductivity properties of the graphene. Notably, there are five factors that influence the sensing applications of graphene: electrical conductivity, surface area, thermal stability, low electrical noise, and mechanical properties [62] (Figure 4b). The electrical conductivity of graphene nanostructures has high carrier mobility and density, even at room temperature, which is advantageous in the fabrication of precious and high-performance electro-analytical devices. In addition, the high surface-to-volume ratio of the graphene allows it to accommodate various receptors for target analytes through van der Waals force, electron transfer, and covalent bonding [63]. Furthermore, the quality of the graphene crystalline lattice can reduce the electrical noise compared with one-dimensional nanostructures. Finally, the mechanical properties of graphene lead to its flexibility and stretchability for use in wearable electronic applications [64]. Sha, R. et al. demonstrated the electrochemical deposition of graphene-polyaniline composites on the glassy carbon electrode (Gr-PANi/GCE) as an affordable method for enzyme-less detection of urea. The observed reduction current in this device shifts towards the positive side during the addition of urea. The electrocatalytic performance of urea at the surface of Gr-PANi/GCE film contains polyanion as an electroactive at neutral pH, whereas Gr flakes, which are highly oxidized, cause a negative charge and play a major role in urea sensing. The decrease in the current due to the interaction between the ions and pi-electrons results in excellent sensitivity and selectivity towards urea [23]. An electrocatalytic material of nickel@carbon nanorod (Ni@CNRs) composite has been prepared by Liu, B.T. et al. using pyrolysis of nickel-based coordination compounds and drop casting on the GCE (Figure 6a–d). The construction of this hybrid structure led to a high surface area along with a homogeneous distribution of Ni on the surface of the structure. Introducing a nickel nanoparticle onto the CNR surface may increase the active region despite its homogeneous distribution. This increase in active region may reduce the distance between Ni nanoparticles and CNRs, thereby enhancing the electron migration rate. These peak (i.e., oxidation current) shifts might be caused by the overlap of the 3d and 4s bands in nickel. In particular, some of the valence electron enter the 3d bands, whereas others enter the 4s bands, as a results holes are generated in the d band. The metal’s ability to receive external electrons during the electrochemical process may be facilitated by the presence of d-band holes. These results show that Ni@CNRs significantly enhance the electrocatalytic performance of an electrode by changing the oxidation potential of uric acid [65]. Electrodeposition of Ni/rGO nanocomposites on the conductive carbon fabric (CCF) was fabricated as a wearable electrode by Singh, A. et al. for the enzyme-free detection of uric acid in sweat (Figure 7a–c). Remarkably, the modified Ni/rGO/CCF electrode can act both as a source of electrons and as a reaction site, and hence it can reduce itself to oxidize the uric acid. This phenomenon is due to the synergetic effect of Ni and rGO on enhancing the electron transfer rate, leading to an increase in the electrocatalytic activity. These results revealed that the Ni/RGO/CCF electrode facilitates the selective detection of UA in human sweat [66]. Naik, T. S. K. et al. studied the hydrothermal method used to make non-enzymatic nickel sulfide (NiS) on graphene (NiS/GO), and the product is further coated on the GC. The presence of GO can enhance the electron transfer rate in the matrix of the working electrode and contributes to the electrochemical sensing of urea (Figure 8a–e). For these things to happen, the possible mechanism is as follows: the potential is applied to Ni^2+^ species that underwent oxidation, followed by the oxidation of Ni^3+^ that can be rehabilitated during the forward scan. This Ni^3+^ is favored to oxidize the urea by converting to the Ni^2+^ state via the electron transfer process. Hence, the fabricated NiS/GO/MGCE electrode enhances the conductivity at the surface electrode interface as a result of the diffusion-controlled process, with and without interference, for the determination of urea [67]. Nia, S. M. et al. prepared nickel-manganese oxo/hydroxo nanoparticles on the GO nanocomposites, which were synthesized via a hydrothermal reduction technique. The obtained Ni(OH)_2_/Mn_3_O_4_/rGO/PANi nanocomposites were used to modify the screen-printed electrodes for the highly sensitive enzyme-less detection of urea. These studies demonstrated that the prepared nanocomposite has the potential to decrease GO functionalities to enhance sensitivity and inhibit the protonation of aniline via graphene structure [68]. Two-dimensional free-standing NiO nanosheets were synthesized using GO paper as a sacrificial template in another study. In the presence of an alkaline medium, electrochemical studies were conducted, which indicated the high stability and electrocatalytic oxidation of urea as well as interferent agents resulting in non-enzymatic sensing of urea. The possible sensing mechanism is that urea can absorb Ni^3+^ ions, which certainly decreases its cathodic peak current. On the other hand, urea would induce the adsorption of oxides intermediated through the active surface sites of the NiO nanosheets. Thus, urea may restrict the kinetics of the redox reaction and cause a positive shift in the anodic peak current that indicates improved sensitivity, quick response time, and good stability [69]. Mohiuddin, A. K. et al. demonstrated the creation of a defect site on nickel-cobalt double-hydroxide decorated graphene (Co_x_Ni_1−x_(OH)_2_/G) using the hydrothermal method. These defect sites can act as superior active sites for the enzyme-free detection of uric acid. The Co_x_Ni_1−x_(OH)_2_ creates a defect and reduces the charge transfer resistance of Ni(OH)_2,_ thus actively participating in the redox reaction. Then, the graphene reduces the aggregation of Co_x_Ni_1−x_(OH)_2_, which improves the electroactive surface region and enhances the electrical conductivity of nanocomposites [70]. The literature also shows that an electrochemical deposition of ITO/PDPA (poly-diphenylamine)/PTA (phosphotungstic acid)/Gra-ME electrode has been designed for its electrochemical activity towards urea detection. Upon increasing the concentration of urea, the oxidation peak current also rises, and the amperometric response of the ITO/PDPA/PTA/Gra-ME-based electrode with the addition of urea shows enhanced electrocatalytic activity. This enhancement is ascribed to the synergistic interaction of sandwiched PDPA/graphene, whereas the tungsten atoms provide fast reversible multi-electron redox behavior that augments the fast electron transfer rate, resulting in improved sensitivity [71]. N-doped graphene nanosheets are prepared by microwave irradiation and subsequently coated onto the glass carbon electrode for the non-enzymatic detection of uric acid in human blood samples (Figure 9a–e). The well-organized oxidation peak is co-related with the higher electrocatalytic activity observed, owing to the nitrogen atom in the N-doped graphene nanosheets interacting with uric acid via a hydrogen bond. This bond may activate hydroxyl and amine groups in uric acid, thus enhancing the electron transfer process using these N-doped graphene [72] nanosheets. A detailed comparison of the literature that pertains to NOC sensors based on graphene and its nanocomposites is given in Table 4.

### 3.3. Field-Effect Transistors (FET)

**Figure 10:**
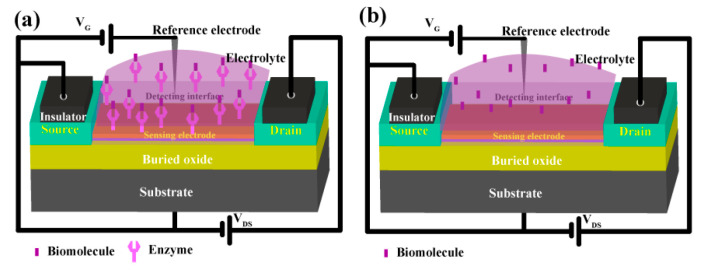
Construction of a field effect transistor (FET)-based device for (**a**) enzymatic and (**b**) enzyme-free detection of NOCs.

**Figure 11:**
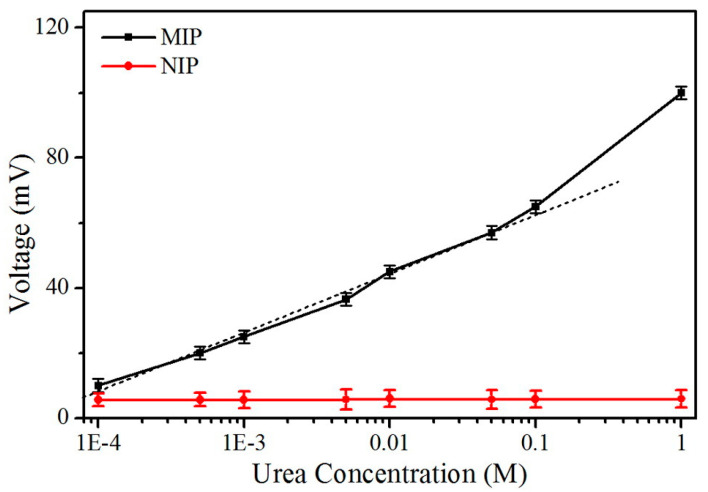
Voltage response of MIP (black line) and NIP (red line) modified ISFET sensors in different urea concentrations (the dotted line is the trend line). Reprinted with permission from reference [80] and the corresponding copyright is 2016 Elsevier.

Using state-of-the-art technology for the fabrication of a low-cost chip-based device called a field-effect transistor (FET) has been demonstrated on a flexible substrate for biosensing applications. Recently, disposable chip sensors have been suggested for real-time diagnosis, in particular, in laboratory-less and non-invasive methods [76]. The field-effect transistor (FET) is an advanced device in which special classes of sensors are used. Biomolecule analysis is one of the many analyses that can be performed using this device, and the device has great use in biomedical applications due to its ability to be a miniaturized device. So, FET requires a very small concentration of specimens, such as serum, blood, or urine [77]. Meanwhile, the electrochemical detection method has been used either by an ion-sensitive electrode or by a field-effect transistor for biochemical sensors (Figure 10a,b). Initially, FET was developed in 1970 by P. Bergveld, followed by its development in 1983 by J. van der Spiegel et al., who first reported an extended-gate ion-sensitive field-effect transistor [78]. In 1997, Pijanowska and Torbicz reported the immobilization of urease on the silicon nitride surface for the detection of urea [79]. In recent times, to fabricate molecularly imprinted polymers (MIP), the photopolymerization method has been considered for constructing an ISFET device, which was developed by Rayanasukha, Y. et al. In this non-enzymatic detection of urea, polymethyl methacrylate (PMMA) and urea are used as polymer membranes and templates, respectively. The MIP-modified ISFET sensor has been optimized by changing features like the thickness of the polymer membrane, the ratio of polymer to urea in the composite, and the incubation time. The electrochemical response of the fabricated MIP-modified electrodes can attain the highest response rate with respect to concentrations (Figure 11). Moreover, the fabricated MIP-modified ISFET possesses high selectivity, good reproducibility, and repeatability. Hence, this ISFET would be a reliable device for the non-enzymatic detection of urea [80]. In addition, a proof-of-concept based SnO_2_:F device was constructed on an FTO-coated glass substrate, which can act as an extended-gate ion-sensitive field effect transistor (EG-FET). Drain-source current (I_DS_) was measured as a function of drain-source voltage (V_DS_) with a fixed Vref voltage. Notably, I_DS_ was measured as a function of Vref with a fixed V_DS_ voltage. The changes in V_FB_ can be indirectly detected with respect to the shift in threshold voltage, which is proportional to the number of charge carriers that have been absorbed over the oxide surface. The enzymatic field-effect transistor sensor response (I_DS_) has been measured with varying urea concentrations, and the voltage has been fixed, and the I_DS_ was measured as a function of time. To determine the sensing performance of the device, the measurements were carried out at different pHs and buffer concentrations. Hence, this proposed method may pave the way for a new class of sensors for clinical diagnosis [81]. A back-gated field effect transistor (BG-FET) was fabricated as a point-of-care (POC) for the detection of urea in a human urine sample. The spin coating method was used to allow the BG-FET to analyze urea. In this method, PMMA is used as a dielectric layer, CdS-TiO_2_ nanocomposites are used as a channel layer, and silver paste is used to make a conductive electrode. The hydrolysis of urea in the presence of urease selectively produces ammonium ions, which were sensed by the BG-FET. As a result, there was an increase in drain current in the BG-FET, with a higher number of charge carriers being generated when ammonia was absorbed on CdS-TiO_2_ nanostructures. To this extent, changes in the measurable signal would be standardized to selectively measure the activity of different types of gas/vapor. The proposed BG-FET sensor design would use real-time, highly sensitive, and selective detection of urea in excretory metabolites, that can be suitable for developing a POC device [82].

### 3.4. Printed Electrodes

**Figure 12:**
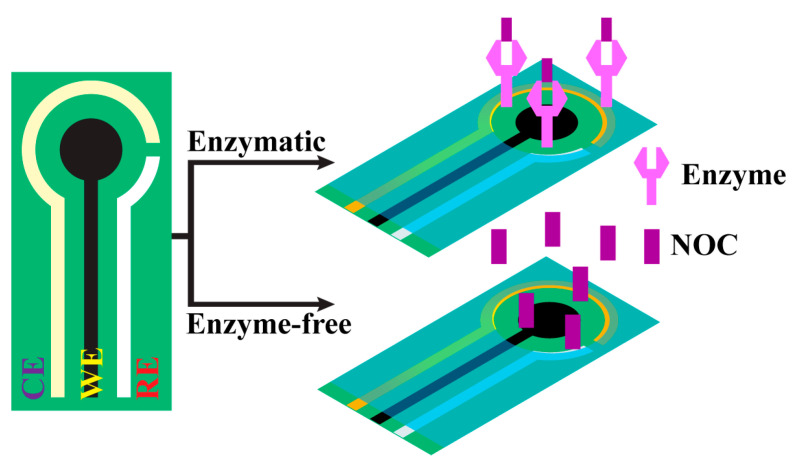
Fabrication of printed electrodes for the detection of NOCs with enzymatic and enzyme-free methods.

As the demand for affordable devices has increased, a simplified fabrication process has been introduced via printing or painting technology to fabricate disposable, flexible, and wearable functional devices [83]. A prototype device has been demonstrated using these techniques for various chemical sensing applications [84]. Nanomaterials contain more active sites at their surface with a high surface area that favors enhanced electron carrier mobility and density. This property is more important in electrically transduced sensing devices. So, printed electrode-based electrically/electrochemically transduced devices have desirable features like affordability, sensitivity, specificity, limit of detection, and reliability (Figure 12) [85]. The first printed electronics were successfully commercialized in household appliances from 1948 to 1960 [86]. In 2011, the first inkjet-printed flexible electronic devices were invented by Massachusetts Institute of Technology (USA) researchers, resulting in more attention among the research community for developing a printed electrode as a device for the detection of various components [87]. In the modern era, metals and metal oxides, carbon derivatives, and conductive polymers are used as ink to fabricate printed electrode-based devices for sensor applications. Hassan, R.Y. et al. studied a conductive polymer/MWCNT nanocomposite coated on the screen-printed electrode (SCE) by drop casting and then used it to detect urea in blood samples. The conductive polymer of poly(o-toluidine) (PoT) was prepared via an oxidative polymerization reaction and was used to fabricate an enzymatic MWCNT/PoT/SCE electrode. The direct electrocatalytic oxidation of the enzymatically produced ammonium ions was obtained using hybrid multi-walled carbon nanotubes and their nanocomposite of PoT (MWCNT/PoT) that can be used for detection of urea in real human blood samples [88]. Electropolymerizing on the molecularly imprinted membrane with a low-cost, flexible urea-PEDOT/c-Au nanotube sensor was used to detect urea via epidermal analysis. The obtained flexible EC sensor was attached to the wrist to absorb urea via sweat, and it was observed that the measurable signal changes as the concentration of urea increases. In human sweat, various substances are present, among which a few have EC activity or are more or less identical in structure to urea. Therefore, it can be a promising method for the efficient, rapid, and non-invasive determination of urea in real sweat samples [89]. The fabrication of urease-MBs/GO/NiO has been performed on the PET substrate using sputtering and screen-printing technology. The electrode surface was functionalized with APTES, and in this, MBs (i.e., magnetic beads) were used to enhance the chemical bonding, thus improving the electrocatalytic performance. The sensing characteristics of urea biosensors have been measured with a voltage-time (V-T) method for MBs/GO/NiO films with low response times, which show excellent charge transition capability. However, there has been a drift effect observed in these biosensors, which might be attributed to the process of adsorption and desorption of the ions and other factors, such as temperature instability, solution contamination, or material decomposition within the sensor, as studied by Chou, J.C. et al. [90]. Berto, M. et al. demonstrated that a fully printed PEDOT: PSS- based organic electrochemical transistor (OECT) has been developed in which the PEDOT: PSS layer acts as a channel and gate layer, respectively. The response of the device is determined by the changes in channel conductivity caused by the ionic species generated during urea hydrolysis catalyzed by the entrapped urease. Screen-printing flexible electrodes using the OECT method is a fascinating technique for large-scale and cost-effective production of point-of-care devices [91]. The screen-printing method, developed by Bao, Q. et al., was used to make a modified multiwalled carbon nanotube/polyaniline (MWCNT/PANi) composite device. The electrical conductivity increased during the polymerization, which was caused by doping with H+. Further, H+ was unbiased from PANi when introducing the urea, resulting in a decrease in the conductivity of PANi and a measurable signal being acquired. With the addition of MWCNT to the PANi chain, there is a formation of conduction channels that causes smooth transduction of electrical signals into measurable signals due to the low conductivity of PANi. Therefore, the modification of the MWCNT/PANi composite significantly enhances the sensitivity for the detection of urea in an enzyme-free method [92]. A detailed comparison of the literature related to NOC sensors made with printable electrodes is given in Table 5.

### 3.5. Electrochemical Impedimetric Detection of NOCs

**Figure 13:**
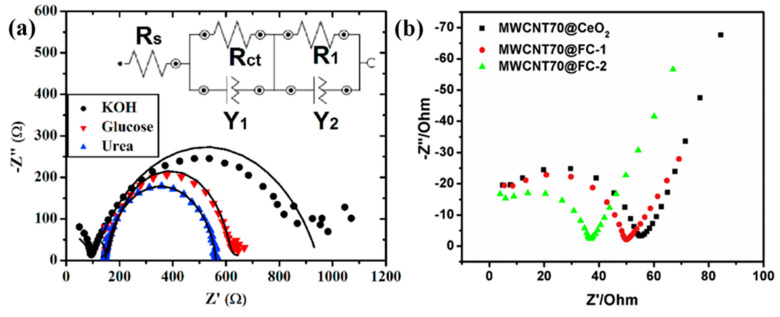
EIS plots of (**a**) NiO–MoO_3_ with the presence of 1 mM urea (blue color) (Inset: Equivalent circuit). Adapted from reference [98] and the corresponding copyright is 2022 Elsevier, and (**b**) the modified GCE with MWCNT70@FC-2 measured in 0.1 M PBS solution containing 0.01 M UA (pH = 6.0) in the frequency region of 1–10 Hz with 50 mV amplitude (Green color). Reprinted with permission from reference [99] and the corresponding copyright is 2020 Elsevier.

Electrochemical impedimetric spectroscopy (EIS) [93] can sensitively monitor changes caused by capacitance or charge transfer resistance during the specific binding of targeted elements [94]. As per the operational principle and mechanism of the measurement, EIS instruments have been differentiated into two types: non-faradaic EIS (i.e., impedimetric transducers) and faradaic EIS (i.e., amperometric and potentiometric transducers). First, the non-faradaic EIS is a non-destructive method as it favors persistent measurement on the same samples. EIS is also performed at various frequency ranges, and it is highly sensitive to even very small changes in the measurement [93,95]. The EIS analysis does not involve any redox reaction and there is no need for direct current or a reference electrode, whereas the faradaic EIS is very focused on electrochemical reactions and provides specific information that does not require multiple frequency sweeps and results in rapid (i.e., less time-consuming) measurements. Faradaic EIS approaches can be employed wherever non-destructive measures are not a priority. So, non-faradaic and faradaic EIS-based detections are more amenable to fabricating a miniaturized kit, in terms of sensitivity, data complexity, and a non-destructive nature [96,97]. Recently, Tammam, R. H. and Saleh, M. M., synthesized a different weight percentage of NiOx on GCE and measured it in an alkaline medium. The fitted Randles equivalent circuit reveals a double-layer capacitor (C_dl_) due to the inhomogeneity and roughness on the surface of the electrode. This capacitor’s presence can be attributed to the increase in the concentration of Ni(OH)_2_ and NiOOH with the increase in the loading percentage. However, R_ct_ decreases due to the faster charge transfer of the redox couple of Ni(OH)_2_/NiOOH, thus converting the fraction of the α-Ni(OH)_2_ species to β-Ni(OH)_2_. Further, this conversion inhibits the diffusion of -OH ions onto the electrode surface. Then, upon increasing the urea concentration, the R_ct_ decreases, which may be attributed to an increase in the charge transfer kinetic due to the higher electrocatalytic oxidation of urea. At higher urea concentrations, the oxidation of Ni (II) to Ni (III) is accelerated. Hence, Ni (III) is removed by urea, which enhances further conversion of Ni (II) to Ni (III), resulting in an increased charge transfer rate caused by the conversion of the OH^−^ ions to CO_3_^2−^ ions and a CO_2_ byproduct [51]. Goda, M.A. et al. demonstrated the R_ct_ measurement for the NiOx/CuOx/PANI/GC electrode during the electrocatalytic oxidation of urea. The fabricated catalyst exhibits a significant decrease in R_ct_, indicating faster charge transfer of NiOx/CuOx/PANI/GC catalysts due to their higher electrocatalytic performance. This may be due to the conductivity contribution of the PANI polymer layer beneath the bimetallic catalyst layer [54]. Salarizadeh, N. et al. developed a NiO-MoO_3_ on GCE, and the EIS results indicate that the R_ct_ value is lower than that of the bare GCE in the presence of urea (Figure 13a). As a result, there was a growth in the reaction rate at the electrode interface. Thus, the decrease in R_ct_ value significantly proves the electroactivity of the developed electrode for the oxidation and enzyme-free detection of urea [98]. Then, electrospun ferric ceria nanofibers were mixed with MWCNTs coated on the surface of GCE by Shekh, M.I. et al. The R_ct_ value was estimated for the MWCNT70@CeO_2_, MWCNT70@FC-1, and MWCNT70@FC-2 tested in the presence of uric acid. Amongst these materials, MWCNT70@FC-2 attained the lowest R_ct_ value for the detection and oxidation of uric acid (Figure 13b). This lowest value is because of the sufficient weight percentage of MWCNT (i.e., 70%) and Fe^3+^ (i.e., 30%) leading to exceptional electrocatalytic performance for the detection of uric acid [99]. Albaqami, M.D. and his colleagues grew Co_3_O_4_ nanowires on cotton silk. Then, they drop-casted uricase onto the Co_3_O_4_ nanostructures on the GCE surface. This fabricated electrode was used to detect uric acid. The fabricated electrode exhibits a higher charge transfer rate, and a small, semicircular Nyquist’s plot of the electrode shows that the nanowires provide a better measurable electrical signal than that of a platelet-like nanostructure. However, immobilization of uricase can slightly influence impedimetrics due to its insulating features in the presence of an enzyme. Hence, the researchers suggested that the proposed nanowire be used for the determination of uric acid because the cotton silk acts as a co-catalyst to boost the electroactive behavior [100].

## 4. Conclusions and Future Perspectives

The development of biosensors using advanced nanomaterials for the detection of NOCs shows desirable sensor characteristics, such as sensitivity, selectivity, limit of detection, and stability. The incorporation of nanoscale materials, such as nickel nanoparticles, graphene, and their nanocomposites, into biosensors has been elaborated. Devices like FET and printed electrodes have revealed the capabilities of a wide range of NOC detection methods and confirmed that extensive and elevated sensing performance has been achieved. The advantages of nanomaterials in the field of sensors make them a new and unavoidable platform for the sensitive analysis of NOCs. These nanomaterials can provide a potential solution not only for biomedical or clinical analysis but also in the fields of agricultural pesticides, environment, and dairy product detection. In this review, we have elaborately discussed the recent advances in the sensing of NOCs using various nanocomposite electrodes with or without functionalization using an enzymatic and/or enzyme-free detection method. A good sensing platform needs to be made as a rapid sensing element that can be used to detect NOCs in real samples in a selective, cheap, disposable, and flexible way. However, the major challenge in this research field is the development of nanomaterials on paper and textile fabrics as microfluidic point-of-care (μ-POC) devices. The state-of-the-art technology in biosensors has been envisioned to construct and develop a selective separation followed by detection of various biomolecules. This detection creates a new roadmap towards the development of affordable, rapid response, wearable, and on-site diagnosis kits in the future.

## Figures and Tables

**Table 1:** The normal NOC levels of urea and uric acid in biofluids [16,17,18,19,20].

S. No.	NOC	Age (Years)	Normal Level (Male) (mg dL^−1^)	Age (Years)	Normal Level (Female) (mg dL^−1^)
1	Urea	1–17	7–20	1–17	7–20
		>18	8–24	>18	6–21
2	Uric Acid	1–10	2.4–5.4	1	2.1–4.9
		11	2.7–5.9	2	2.1–5.0
		12	3.1–6.4	3	2.2–5.1
		13	3.4–6.9	4	2.3–5.2
		14	2.7–7.4	5	2.3–5.3
		15	4.0–7.8	6	2.3–5.4
		>16	3.7–8.0	7–8	2.3–55
				9–10	2.3–5.7
				11	2.3–5.8
				12	2.3–5.9
				>13	2.7–6.1

**Table 2:** The normal NOC level of creatinine in biofluids [21,22].

S. No.	NOC	Age (Years)	Normal Level (Male)	Age (Years)	Normal Level (Female)
1	Serum creatinine	19–75	0.74–1.35 mg dL^−1^	19–75	0.59–1.04 mg dL^−1^
	Typical range based on BSA *	19–75	77–160 mL/min/BSA	18–29	78–161 mL/min/BSA
				30–39	72–154 mL/min/BSA
				40–49	67–146 mL/min/BSA
				50–59	62–139 mL/min/BSA
				60–72	56–131 mL/min/BSA
2	Albumin/creatinine ratio #	19–75	<17 mg/g	19–75	<25 mg/g

* BSA—Body surface area, # Increase in the ratio above this level could be a sign of kidney disease.

**Table 3:** Comparison of electrochemical detection of urea using metal oxides and their nanocomposites.

S. No	Electrode Type	Analytical Technique	Enzyme Immobilization Method	Linear Range	Limit of Detection	Real Samples	Ref.
1.	Ni-P	Amperometric	Enzyme free	0.05–11 mM	12 µM	Swimming pool water	[50]
2.	Co-ZIF-NiMWs	DPV	Enzyme free	0.0005–0.5 mM	0.30 µM	Human urine and milk	[52]
3.	NiO/cESM/GCE	SWV	Enzyme free	0.05–2.5 mM	∼20 µM	Tap water	[53]
4.	NiCo_2_O_4_ NWs/GCE	CV	Enzyme free	0.01–5 mM	1.0 µM	-	[55]
5.	Ni(OH)_2_/GCE	CV and DPV		25–90 µM	1.701 µM	-	[56]
6.	Ur/NiO/ITO/glass	CV	Enzymatic	0.83–16.65 mM	0.28 mM	-	[57]
7.	NiO/cellulose/CNT	Chronoamperometric	Non-enzymatic	0.01–1.4 mM	7 µM	Urine	[42]
8.	Vitamin C based NiO/GCE	Amperometry	Non-enzymatic	100–1100 μM	10 μM	Mineral, river, and tap water	[58]
9.	NiO/CTAB/GO/GCE	Amperometry	Non-enzymatic	100 –1200 µM	8 µM	Mineral, river, and tap water	[59]
10.	Ni/Au electrode	CV	Non-enzymatic	-	3.35 × 10^−2^ mM	Urine	[60]

**Table 4:** Comparison of electrochemical detection of urea using graphene and its nanocomposites.

S. No	Electrode Type	Analytical Technique	Enzyme Immobilization Method	Linear Range	Limit of Detection	Real Samples	Ref.
1.	Gr-PANi/GCE	I–V	Non-enzymatic	10–200 μM	5.88 μM	Milk and tap water	[23]
2.	Ni@CNRs	DPV	Enzyme free	35–100 μM	0.166 μM	Human urine	[65]
3.	Ni/RGO/CCF	DPV	Enzyme free	10–60 μM	5.083 μM	Human sweat	[66]
4.	NiS/GO/MGCE	CV	Enzyme free	0.1–1.0 mM	3.79 μM	Milk	[67]
5.	Ni(OH)_2_/Mn_3_O_4_/rGO/PANi	CV	Enzyme free	30 μM–3.3 mM	16.3 μM	Human serum	[68]
6.	2D NiO papers	i-t	Enzyme free	4.4–181.6 mM	2 μM	-	[69]
7.	CoxNi1−x(OH)_2_/G/GCE	DPV	Enzyme free	0.25–925 μM	0.097 μM	Urine	[70]
8.	ITO/PDPA/PTA/Gra-ME nanohybrid	CV	Enzyme free	1–13 μM	-	-	[71]
9.	NG	CV	Enzyme free	0–600 μM	0.0045 μM	Serum	[72]
10.	GND/PANI/urease	I–V	Enzymatic	0.1–0.9 mg mL^−1^	0.05 mg mL^−1^	-	[73]
11.	Graphene nanoplatelet/graphitized nanodiamonds nanocomposite	I–V	Enzymatic	0.1–0.9 mg mL^−1^	5 μg/mL	-	[74]
12.	NiCo_2_O_4_/3D graphene/ITO	Chronoamperometric	Non-enzymatic	0.06–0.30 mM	5.0 µM	Urine	[75]
13.	NiS/GO/MGCE	CV	Non-enzymatic	0.1–1.0 mM	3.79 µM	Milk	[67]

**Table 5:** Comparison of electrochemical detection of urea using printed electrode devices.

S. No	Electrode Type	Enzyme Immobilization Method	Linear Range	Limit of Detection	Analytical Technique	Real Samples	Ref.
1.	MWCNT/PoT/SPE	Enzymatic	0.1–11 mM	0.03 mM	CV	Human blood	[88]
2.	PEDOT/C-Au NTs EC	Non-enzymatic	1−100 mM	-	DPV	human sweat	[89]
3.	Urease/MBs/GO/NiO	Enzymatic	1.665–8.325 mM	0.223 mM	V-T	-	[90]
4.	OECTs	Enzymatic	1 μM–10 mM	1 μM	I-V	-	[91]
5.	MWCNT/PANi-modified SPCE	Non-enzymatic	10–50 µM	10 µM	CV	-	[92]

## Data Availability

No new data were created or analyzed in this study. Data sharing is not applicable to this article.
